# Medical Management of Parkinson’s Disease: Focus on Neuroprotection

**DOI:** 10.2174/157015911795596577

**Published:** 2011-06

**Authors:** Marie-Catherine Boll, Mireya Alcaraz-Zubeldia, Camilo Rios

**Affiliations:** 1 Department of Clinical Investigation in Neurology National Institute of Neurology and Neurosurgery, Mexico. D.F.; 2 Department of Neurochemistry National Institute of Neurology and Neurosurgery, Mexico, D.F.

**Keywords:** Parkinson´s disease, neuroprotection, clinical trial, rasagiline, pramipexole.

## Abstract

Neuroprotection refers to the protection of neurons from excitotoxicity, oxidative stress and apoptosis as principal mechanisms of cell loss in a variety of diseases of the central nervous system. Our interest in Parkinson’s disease (PD) treatment is focused on drugs with neuroprotective properties in preclinical experiments and evidence-based efficacy in human subjects. To this date, neuroprotection has never been solidly proven in clinical trials but recent adequate markers and/or strategies to study and promote this important goal are described. A myriad of compounds with protective properties in cell cultures and animal models yield to few treatments in clinical practice. At present, markers of neuronal vitality, disease modifying effects and long term clinical stability are the elements searched for in clinical trials. This review highlights new strategies to monitor patients with PD. Currently, neuroprotection in subjects has not been solidly achieved for selegiline and pramipexole; however, a recent rasagiline trial design is showing new indications of disease course modifying effects. In neurological practice, it is of utmost importance to take into account the potential neuroprotection exerted by a treatment in conjunction with its symptomatic efficacy.

## 1. INTRODUCTION

Although multiple neuronal systems are involved, the hallmark of Parkinson’s disease (PD) pathology is centered on cell loss in *substantia nigra pars compacta* (SNc) directly affecting the dopaminergic nigro-striatal pathway. Strategies to monitor dopamine nerve terminals *in vivo* in PD brains include striatal uptake of (18)fluorodopa or (11)C-beta-CFT [2-beta-carbomethoxy-3beta-(4-fluorophenyl) tropane] revealed, by positron emission tomography (PET) [1, 2, 3] and beta-CIT or (123)I in single-photon emission computerized tomography (SPECT), to show distribution of dopamine transporters (DAT), among other tracer-DAT ligands [4, 5]. However, it has not yet been confirmed that striatal uptake of these isotopes does in fact correlate with the remaining number of dopamine neurons or terminals. Furthermore, the possibility of a confounding pharmacological effect has not yet been completely excluded [6]. Vesicular monoamine transporter-2 (VMAT-2) and Nurr1 are two other surrogate markers of DA neuron function whose expression is still being studied in cell cultures and tissues [7, 8]. Based on the possible interventions available for protecting vulnerable neurons, different strategies and treatment algorithms have been presented [9, 10]. However, until now, no drug has yet been established as a neuroprotective agent in human subjects, and none has yet been approved for neuroprotective indication. In 2003, twelve potentially neuroprotective compounds were prioritized by a large expert group, the CINAPS (NIH- appointed Committee to Identify Neuroprotective Agents in Parkinson´s; for consultation, see Table **1**), to be further studied in clinical trials [11]. We present here the mechanisms studied and the actual status of those compounds, as well as new drugs with protective properties that are currently being used in human subjects. The order of presentation provided here is based on the chronologic neuroprotective focus attributed to a compound or class of drug in clinical practice.

## 2. COMPOUNDS OR CLASS OF DRUGS PRIORITIZED FOR THEIR NEUROPROTECTIVE PROPERTIES

### 2.1. MAO-B Inhibitors

#### 2.1.1. Selegiline 

Among the drugs that have received most of the attention in relation to neuroprotection, selegiline (deprenyl) and rasagiline stand out, which are monoamine oxidase type-B (MAO-B) inhibitors. Both of these compounds of the propargylamine group have demonstrated neuroprotective efficacy in cell culture and animal models. Selegiline not only protects cultured dopaminergic neurons against MPP+ (1-methyl-4-phenylpyridine), the toxic metabolite of MPTP (1-methyl-4-phenyl-1, 2,3,6-tetrahydropyridine) [12], but also reduces the cell death cascade caused by MPTP in animal models [13]. These benefits were initially thought to depend only on monoamine oxidase B inhibition through prevention of excessive production of reactive oxygen species (ROS) by catabolism of monoamines, but it is now known that selegiline also enhances the synthesis of neurotrophic factors [14]. This compound can also protect against apoptotic cell death, possibly *via* an increased production of Bcl-2, a member of a protein family that modulates the release of cytochrome c into the cytoplasm and the resulting destructive cascade of compounds [14, 15, 16], or by maintaining glyceraldehyde-3-phosphate dehydrogenase (GAPDH) as a dimer, thereby preventing its nuclear translocation and its blocking upregulation of anti-apoptotic proteins [13, 14, 17].

The DATATOP (Deprenyl and Tocopherol Antioxidant Therapy of PD) study is the most important clinical trial for demonstrating the beneficial effects of deprenyl in PD patients. From 1987 to 1988, a cohort was conformed of 800 patients harboring early Parkinson’s disease. Subjects were randomized to receive deprenyl, tocopherol, combined treatments, or a placebo. The study showed that deprenyl (10mg/d) significantly delayed the onset of disability requiring levodopa therapy [18]. A second independent randomization was carried out in 1993 with 368 subjects, who, by that time, had required levodopa and who had consented to continue the study by adding deprenyl or placebo assigned under double-blind conditions to their treatment. The first development of a levodopa complication, such as the wearing off phenomenon, dyskinesias, or on-off motor fluctuations, was the primary outcome measure. During the average 2-year follow-up, there were no differences between treatment groups with respect to the primary outcome measure, withdrawal from the study, death or adverse events. Subjects under deprenyl developed more dyskinesias (34% *vs*.19% under placebo; p=0.006) and significantly less freezing of gait (16% *vs*. 29%; p=0.0003). Decline in motor performance was also less in subjects under deprenyl treatment. Levodopa-treated PD patients who had received deprenyl for up to 7 years, compared with patients who were changed to a placebo after about 5 years, experienced slower motor decline and, additionally, were more likely to develop dyskinesias but less likely to develop freezing of gait [19]. An evaluation of the DATATOP cohort in subjects followed-up for two decades did not confirm the expected neuroprotection, since the interpretation of the results was confounded by a drug symptomatic effect [5]. The SIN-DEP-PAR (Sinemet-Deprenyl-Parlodel) study [20] used the change in motor score between initial visit and final visit after elimination of all study medications as the primary end point. However, here, too, there were concerns about confounding symptomatic effects, since several antiparkinsonian medications were shown to have a long duration response that could persist for weeks and perhaps even months after withdrawal. Drugs that have already been approved in PD for their symptomatic effects, such as dopamine agonists or propargylamines, offer the best opportunity for establishing the neuroprotective feature of a drug, even if the observed benefits lie within their symptomatic properties. Currently, this will most likely require demonstrating that the drug provides benefit for PD patients using both imaging and clinical markers of disease progression [6, 17, 20]. As postural hypotension, a known side-effect of L-DOPA treatment, may be potentiated when L-DOPA is combined with selegiline, other IMAO-B drugs lacking the sympathomimetic effects of selegiline seem to be indicated in subjects presenting this adverse event.

#### 2.1.2. Rasagiline

Lately, rasagiline (N-propargyl-1 (R)-aminoindan) has demonstrated to be a highly selective and irreversible potent inhibitor of MAO-B with properties similar to those described for selegiline with the exception that, unlike selegiline, rasagiline is an aminoindan derivative with no amphetamine metabolites. Two important trials in PD patients, TEMPO (TVP-1012 in Early Monotherapy for Parkinson's Disease Outpatients) and LARGO (Lasting Effect in Adjunct Therapy With Rasagiline Given Once Daily), are successors of many preclinical studies that have demonstrated protective effects, *in vitro* as well as *in vivo*, against a wide range of neurotoxins [21]. Aminoindan is, by itself, neuroprotective in cultured PC-12 cells that, in absence of serum and nerve growth factor (NGF), die after an apoptotic process [22]. Rasagiline was shown to increase survival in a Cu/Zn SOD transgenic mouse model of ALS [23] and was the first with demonstrated efficacy, as initial monotherapy, in the large TEMPO study. Four hundred and four patients with early PD not requiring dopaminergic therapy were randomized to receive 1mg or 2mg once daily of oral rasagiline or placebo. Both dosages in this trial were effective in relation to placebo and the difference between 1 and 2 mg was not significant [24]. In the extension phase of the TEMPO trial, patients initially under placebo also received rasagiline. As a result, the functional decline was significantly more pronounced in patients whose treatment was deferred. Delayed-start analysis of the TEMPO study suggests that rasagiline may slow the rate of progression of PD [25]. In fact, in the recent ADAGIO study, patients who received 72 weeks of treatment exhibited significant clinical differences with those receiving rasagiline for only the last 36 weeks [26]. The rate of progression in both groups was graphically represented by parallels, and the slopes never converged during the entire follow-up. The authors of this study maintain that the difference between groups illustrates the neuroprotection conferred by early treatment in contrast to that provided by the delayed start.

### 2.2. Caffeine

Epidemiological studies have firmly established the relationship between the consumption of caffeine and a low risk of PD [27]. Adenosine A_2A _receptor is a G protein–coupled type of ligand binding protein, abundant in basal ganglia and a target of caffeine. Adenosine A_2A _receptor antagonists and caffeine provide a broad spectrum of neuroprotection in different animal models of PD [28, 29] through the modulation of glutamate release and inflammation in the CNS [30]. KW6002, the adenosine A_2A _receptor antagonist tested in many PD models, was found to enhance motor activity even in dopamine-depleted animals. Further named istradefylline, it was recently shown to improve motor function in patients with Parkinson´s disease [31].

### 2.3. Estrogen

Most animal studies support the ability of estrogen to function as a neuroprotective against neurotoxins that target the nigrostriatal dopaminergic system [32]. Glia-neuron crosstalk has been shown to play a cardinal role in directing neuroprotection *vs*. neurodegeneration, in addition to the specific role exhibited by astroglia in modulating inflammation in estrogen neuroprotection, manifested as a variable response of astrocytes and microglia to MPTP injury according to estrogenic status [33]. Other potentially protective properties are derived from the co-localization of estrogen receptors to cells that express BDNF and its receptor trkB, as well as the further regulation of the expression of this neurotrophin system [34]. Activation of membrane estrogen receptors also induce pro-survival kinases leading to CREB phosphorylation and NFκB nuclear translocation, two transcription factors controlling the expression of anti-apoptotic Bcl-2 proteins [35]. The relationship between the structure and the estrogenicity of flavonoid derivatives explains their protection on stressed dopaminergic neurons through the inhibition of microglial activation [36]. In clinical studies, estrogen therapy replacement in women has not only been associated with improved scores in the UPDRS (Unified Parkinson Disease Rating Scale) [37], but also with the reduction of peak-dose dyskinesias [38]. Meanwhile, oöphorectomy before the onset of menopause exhibited an increased risk of Parkinsonism, although this risk was generally similar in women using hormones and in those who had never used them [39]. However, estrogens were associated with a reduced risk of PD among women with low caffeine consumption, suggesting that caffeine, protective in men, reduces the risk of PD among women not using postmenopausal hormones, but increases the risk among hormone users [40].

### 2.4. Minocycline

Tetracyclines possess a wide range of antimicrobial activity that works by exerting the inhibition of bacterial protein synthesis. Initially evaluated in ischemic tissues, minocycline, a more lipophylic tetracycline derivative, has remarkable neuroprotective properties. It has been demonstrated that minocycline inhibits ischemia and induces up-regulation of nitric oxide synthase, caspase 1 and reactive microgliosis [41-43]. Neuroprotection by minocycline has also been observed in HD and ALS transgenic mouse models [44, 45]. A primary mechanism has been proposed in which the release of cytochrome c is inhibited and the formation of apoptosome in the cytoplasm and downstream can cause a “contagious apoptosis”, since toxic factors generated by dying neurons can affect neighboring cells [46]. In the PD mouse model, minocycline attenuates MPTP-induced dopaminergic neurodegeneration and decreases MPTP-mediated nitrotyrosine formation and microglial activation. Minocycline confers resistance to the MPTP toxin by preventing the production of deleterious microglial-derived mediators, such as proinflammatory cytokine IL-1β, nitric oxide and ROS [47]. But, other than current experimental evidence for the potential use of minocycline in PD, there are still few clinical assays and no clinical evidence of its neuroprotective properties in PD patients. To this date, we can only mention one futility clinical trial of creatine and minocycline in early PD. Futility studies are phase II trials designed to eliminate drugs showing low potential, hence avoiding further large and expensive phase III studies. Taking as futility threshold the mean change in UPDRS in the placebo/tocopherol arm of the DATATOP study, neither minocycline nor creatine could be rejected as futile for further trials [48].

### 2.5. Creatine

This guanidine derived compound is converted to phosphocreatine, which serves to transfer phosphoryl groups in mitochondrial ATP synthesis. Creatine also reduces oxidative stress through the stabilization of mitochondrial creatine kinase and the consequent opening of the mitochondrial transition pore that activates apoptosis [49]. A few experimental studies suggest a neuroprotective role of dietary intake of creatine in PD models. It is now considered for phase III by NINDS NET-PD Investigators, as the phase II futility study showed even better clinical results in the creatine group than in the minocycline one [48]. The study is currently recruiting participants for the creatine arm NET-PD LS-1.

### 2.6. Coenzyme Q10

Several other agents have shown to be beneficial in PD animal models, such as, coenzyme Q10, ginkgo biloba, nicotinamide, and acetyl-L-carnitine [50]. Among them, coenzyme Q10, an essential cofactor of the electron transport chain as well as a potent antioxidant, has been prioritized [11]. A specific defect of Complex I (NADH-ubiquinone reductase ) in the mitochondrial respiratory chain has been described in the *substantia nigra* of patients with Parkinson's disease, similar to the effect of MPTP in those animal models that show specific destruction of the dopaminergic neurons in the *substantia nigra*. Coenzyme Q10 is able to attenuate this dopaminergic neuron induced loss through free radical scavenging and the specific regulation of the mitochondrial permeability transition pore [51, 52]. Although the serum levels of CoQ10 are normal in patients with Parkinson's disease, the administration of 300 mg, 600 mg and 1200 mg CoQ10/d, respectively, showed a significant trend relating to dosages and mean change in UPDRS [53]. Safety and tolerance were found at the highest doses tested -2400 and 3000 mg/d- when the plasma level plateau is reached [54].

These effects were further tested in a phase II clinical trial together with another promising drug: GPI-1485. The results of this study indicate that both drugs should be considered for phase III studies.

### 2.7. GPI-1485

This compound is a neuroimmunophilin ligand which acts by binding the intracellular receptor-protein, in a manner similar to some immunosuppressive drugs. Immunophilin ligands can be ingested orally and may promote nerve growth in a better fashion than neurotrophic factors [55] without clinical immunosuppressive activity. 

The futility study was carried out with a threshold taken from the historical controls of the DATATOP study [18] with the indication that both drugs should be considered for phase III clinical trials. Nevertheless, using recent controls in both minocycline-creatine and coenzyme Q10- GPI-1485 phase II studies, only creatine can still be considered a promising candidate for future studies, probably due to the marked low rate of change in UPDRS exhibited during a one-year interval in all groups [56].

### 2.8. GM1 Ganglioside

Since the early 1980s, numerous studies have documented the beneficial effects of GM1 ganglioside in different brain damage models. In addition to rescuing damaged dopamine neurons, when administered intra peritoneally, GM1 was found to enhance the synthesis of dopamine in remaining nigrostriatal neurons following MPTP exposure [57]. In PD patients, anti-GM1 ganglioside antibodies have been found, especially in those presenting a tremor-dominant form of the disease [58]. The last clinical trial of efficacy of GM1 [59], despite manifesting safety and clinical improvement, did not have any continuity, probably due to the problem of bioavailability of the oral form and the requirement of intravenous or subcutaneous injections.

### 2.9. Dopamine Agonists

Dopamine receptors, members of a super family of G protein-coupled receptors, play a crucial role in mediating the wide array of effects of dopamine in the CNS, especially in the limbic system and the motor control pathway. The five molecular human subtypes are divided into 2 subfamilies: D1-like receptors; D1 being the predominant form and D5, and D2-like receptors; D2 being highly expressed, and D3 and D4 rare. The division is based on their capability to stimulate adenylate-cyclase (D1-like) or to inhibit this enzyme (D2-like). D1 and D2 receptors, mainly found in the *striatum* and *substantia nigra*, are localized postsynaptically. D2 also acts presynaptically as an autoreceptor, modulating synthesis and release of dopamine, while D3, other autoreceptor, may control basal dopamine levels in the striatum without regulating dopamine transporters or tyrosine hydroxylase [60]. Currently used in clinical practice, DA agonists include the ergot-derivatives bromocriptine, cabergoline, dihydroergocriptine (DHEC), lisuride and pergolide, as well as the non-ergot compounds apomorphine, piribedil, pramipexole, ropirinole and rotigotine. This distinction raises the question whether differences exist in their pharmacological properties, therapeutic effects and safety. It is generally accepted that ergolines may cause retroperitoneal and pleuropulmonar fibrosis (rarely) and valvulopathies in 30% of patients under treatment with pergolide. Probably, other ergot-derivative DA agonists such as cabergoline may cause the same severe adverse events [61]. As cardiovascular risk factors could represent confounders or cofactors, performing an echocardiography before initiation of treatment with a dopamine agonist is recommended [62]. The exact pathway leading to valvulopathy is unknown, although agonism of 5-HT(2B) receptors in the heart has been implicated as a mediator in the process. Other ergolinic dopamine agonists, such as lisuride, and non-ergot dopamine agonists with no 5-HT(2B) agonistic activity, may not induce fibrotic changes in heart valves. Given the clinical consequences of the adverse reactions, affinity for 5-HT(2B) receptors must be routinely tested in future drugs [63]. One interesting finding drawn from clinical trails is that four chemically different DA agonists: ropirinole, pramipexole, cabergoline and pergolide delay the necessity of levodopa treatment in PD patients and also decrease the occurrence of prolonged L-DOPA treatment complications [64]. The treatment of early Parkinson's disease with bromocriptine may also be beneficial in delaying motor complications and dyskinesias with comparable effects on impairment and disability in those patients that tolerate the drug bromocriptine versus levodopa in early Parkinson's disease [65].

#### 2.9.1. Pramipexole

Pramipexole is a dopamine D2/D3 receptor agonist used to treat Parkinson's disease. Both human and animal studies suggest that pramipexole may exhibit neuroprotective properties involving dopamine neurons. However, the mechanisms underlying its neuroprotective effects remain uncertain. The results of Pan *et al*. [8] reveal a novel cellular action of this agent. Specifically, pramipexole rapidly increases vesicular dopamine uptake in synaptic vesicles prepared from *striata* of treated rats. This effect presents the following characteristics: 1) it is associated with a redistribution of vesicular monoamine transporter-2 (VMAT-2) immunoreactivity within nerve terminals, and 2) it is prevented by pre-treatment with the dopamine D2 receptor antagonist, eticlopride. The implication of these findings is relevant to the treatment of several neurodegenerative disorders involving the dopaminergic system [4, 66, 67].

#### 2.9.2. Ropirinole 

Another D2/D3 dopamine agonist available in some countries, ropirinole, besides providing preclinical evidence of neuroprotective properties, has also shown a slower rate of decline of functional markers in 18 F-DOPA PET-scans, compared to levodopa [68]. No difference in the progression of motor impairment has been found between new agonists and levodopa [69].

Possible side effects of all DA agonists are nausea, vomiting, hypotension, leg-edema, dizziness and bradycardia, due to autonomic peripheral stimulation. Co-administration of a peripheral DA receptor blocker, domperidone, can counteract these symptoms. Central side effects -such as somnolence [70] and hallucinations- are less frequent with carbidopa/levodopa as initial treatment but the advantages of new agonists -such as pramipexole and ropirinole- are possible neuroprotection and lower frequency of dyskinesias and motor fluctuations [71]. DA agonists as adjuncts to levodopa generally reduce motor fluctuations by increasing “ON” time and reducing “OFF” time [72]. Low acting agonists -cabergoline, piribedil, DHEC and pergolide- do not generally induce dyskinesias in primate models [73]. L-DOPA induced dyskinesias are also improved by high doses or continuous subcutaneous infusion of apomorphine or lisuride, with results similar to those of levodopa intravenous and enteral (duodenal/jejunal) infusion [74]. A transdermal patch formulation of rotigotine, the non-ergolinic D3, D2, D1 receptor agonist, is now being proposed for use as monotherapy in the treatment of early-stage Parkinson's disease as an adjunct to levodopa [75].

## 3. NEW PROMISING DRUGS FOR NEUROPROTECTION IN PD-PATIENTS

### 3.1. Istradefylline

This adenosine A_2A _receptor antagonist (like caffeine) exerts motor effects at a daily dose of 40 mg now reported in a clinical trial [31]. Other good prospects for PD therapeutics are presented in Table **2**.

### 3.2. Zonisamide

This drug, 1,2-benzisoxazole-3-methanesulfonamide, is indicated as an adjunct therapy for partial seizure disorders. Zonisamide acts by blocking voltage gated sodium and calcium channels. It modulates central dopaminergic, GABAergic and serotonergic pathways, and inhibits carbonic anhydrase and monoamine oxidase B. Therefore, it is potentially efficient in an array of other neurological disorders including migraine and other headache syndromes, neuropathic pain, Parkinson's disease, essential tremor and stroke [76]. A multicenter, randomized, double-blind, placebo-controlled study was recently conducted in Japanese PD patients, showing significant improvement in the motor subscale of the UPDRS with 25 and 50 mg /d as adjunctive treatment to levodopa therapy [77]. Effects of zonisamide against DA quinone formation could be protective since DA quinones conjugate with several key PD pathogenic molecules (e.g., tyrosine hydroxylase, alpha-synuclein and parkin) to form protein-bound quinone (quinoprotein) and consequently inhibit their functions [78].

### 3.3. Safinamide

A new agent, safinamide, a monoamine oxidase-B and glutamate release inhibitor, is now in phase III development. Although not yet approved, safinamide may offer the added advantage of dopamine reuptake inhibition. Early reports confirm the potential efficacy of this unique molecule in PD, although studies on its effects on cognition and neuroprotection are needed [79, 80].

### 3.4. Resveratrol

Sirtuins, a family of conserved proteins with deacetylase and ADP-ribosyltransferase activities, are other good prospects in PD therapeutics. The most widely investigated and best known sirtuin is SIRT1. The human gene corresponds to yeast sir-2. Enhancement of SIRT 1 and reduction of SIRT2 expression delay the toxic effect induced by α-synuclein, the protein that forms insoluble aggregates in dying dopamine neurons [81]. SIRT1, which can be activated by the natural phytocompound resveratrol, plays a role in several physiologic conditions ranging from gene silencing, control of the cell cycle, apoptosis and energy homeostasis. A famous study of the role of sirtuins on the extension of life-span in caloric restriction models [82] was rapidly followed by the commercialization of several presentations of reverastrol capsules, before having clearly stated the purity percentage which indicates the contribution of trans-R (the active form) in relation to total product weight. Those presentations are promoted in many dietary and well-being management programs. In the field of neurodegeneration, resveratrol has proved to be beneficial in *in vitro* and *in vivo* models of Alzheimer's disease (AD) by reducing amyloid-beta protein accumulation [83]. So far, no work on PD has been published, but recent literature strengthens the perception that diverse molecular signaling pathways are participating in the neuroprotective activity of many phytocompounds, and a new trial with green tea polyphenols is now recruiting PD patients. 

## 4. OLD DRUGS WITH NEW PROPERTIES

### 4.1. Amantadine

In 1968, a 58-year-old female patient reported that her Parkinson’s symptoms had decreased while she was taking amantadine to prevent the flu. The first trials with this drug in PD showed a peak effect during the first week with one half of the patients showing improvement [84]. Moreover, the beneficial effects were definite but usually brief. After three decades of empiric use of amantadine in PD, the drug was finally characterized as a noncompetitive NMDA (N-methyl D-aspartate) receptor antagonist [85, 86]. When activated, the NMDA receptor, a specific type of ionotropic glutamate receptor, plays a key role in a wide range of physiologic and pathologic processes, such as calcium-mediated excitotoxicity. A 2001 Cochrane review pointed out in its conclusions that the considerable amount of evidence supporting the effectiveness of amantadine was accrued from non-controlled trials, often in patients with Parkinson’s condition other than idiopathic PD and that a rigorous analysis of the six controlled trials revealed insufficient evidence of its efficacy in the disease [87]. Since then, like its congener memantine, amantadine showed improvement of both levodopa induced dyskinesias and PD subcortical dementia [88]. Additionally, an amantadine treatment and its duration were significantly correlated with a late onset of dementia as well as attenuation of its severity in PD patients [88].

### 4.2. Valproic Acid 

Valproic acid (VPA), an established antiepileptic and mood-stabilizer drug, has recently emerged as a promising neuroprotective agent. This aliphatic acid compound represents with the orphan drug phenylbutyrate a class of histone deacetylase (HDAC) inhibitors. Moreover, VPA has been shown to mediate neuronal protection by activating signal transduction pathways and inhibiting proapoptotic factors. In a cerebral hemorrhage model, VPA inhibited caspase activities. In addition, inflammatory cell infiltration was associated with enhanced histone H3 acetylation, expression of extracellular signal regulated kinases, CREB, heat shock protein HSP70, Bcl-2 and Bcl-xl, whereas the expression of Fas-L (a proapoptotic factor), matrix metalloproteinase-9 and interleukin 6 (IL-6; marker of inflammation) were down-regulated [89]. In an animal model of PD, in which degeneration of nigro-striatal dopaminergic neurons is obtained through sub-chronic administration of rotenone, a mitochondrial toxin, a treatment with VPA counteracted the death of nigral neurons and the 50% drop of striatal dopamine levels as well as the alpha-synuclein alterations caused by rotenone [90]. No results in PD patients are still available. 

### 4.3. Lithium Carbonate

Lithium has been used for six decades to treat mania, the up phase of bipolar disorder. Lithium ions interfere with the sodium pump that relays and amplifies messages carried to the cells of the brain and act by inhibiting protein kinase C (PKC) activity. Daily doses of lithium have been found to delay progression of ALS in a recent clinical trial performed in a small group of patients [91]. In the same report, an experiment in SOD1 transgenic mice reported decreased ubiquitin, SOD1 and alpha-synuclein aggregation in Litreated animals. Also, mitochondrial vacuolization and astrocytic proliferation were decreased under lithium treatment. Finally, the most important finding reported in this experiment was the marked increase in autophagy vacuoles in motoneurones from treated animals. Autophagy is a critical pathway that regulates the clearance of aggregated proteins among diverse intracellular pathogens. Lithium and valproic acid, by acting on the mTOR-independent pathway, enhance autophagy and are therefore protective in different neurodegenerative models [92]. To this day, Li is still not indicated for the treatment of PD, but, interestingly, a robust synergistic protective effect of Li and VPA used in combination has recently been described in an ALS mouse model [93]. Glutamate mediated excitotoxicity as well as glycogen synthase kinase 3 (GSK-3) activity were significantly decreased by this combined treatment.

### 4.4. Copper Sulfate

Copper is an essential trace element present in serum and bound to ceruloplasmin (93% of total serum copper) or albumin (7% of total serum copper). Since copper ions adopt distinct redox states, they play an important role in cell physiology as cofactors in the redox chemistry of enzymes involved in a variety of biological activities, such as complex IV of mitochondrial respiratory chain (cytochrome c oxidase) and other powerful free radical scavengers (metallothionein, superoxide dismutase and ceruloplasmin, among others). The neurodegenerative phenotypes of Menkes’ and Wilson’s diseases underscore not only the essential nature of this metal in nervous system development, but also the consequences of the unbalanced neuronal copper homeostasis. Moreover, inherited deficiency of ceruloplasmin is associated with progressive retinal and basal ganglia neurodegeneration [94]. Recent studies have also indicated the involvement of copper in the pathogenesis of Alzheimer’s disease, prion-mediated encephalopathy, amyotrophic lateral sclerosis and PD. Our results showed that increased levels of free copper in the CSF seem to be a good marker of several neurodegenerative diseases, but mainly PD [95]. These findings have been related to the reduced ferroxidase activity of brain ceruloplasmin and increased iron deposition in SNc. Copper acts as a prosthetic group of several copper-dependent antioxidant enzymes. When administered in a mouse model of PD as a pretreatment, CuSO_4_ showed its neuroprotective effect against the administration of 1-methyl-4-phenylpiridinium (MPP+), the toxic metabolite of 1-methyl-4-phenyl-1,2,3,6-tetrahydropyridine (MPTP), by blocking lipid peroxidation. The dose and time dependent protection observed have been associated with the safeguarding of striatal DA levels [96]. In the same model, a specific and significant increase in manganese superoxide dismutase activity was found under copper treatment [97]. Copper pretreatment also prevented tyrosine hydroxylase inactivation by blocking protein nitration in rats [98] and mice [99]. These results were accompanied by a significant reduction in enzymatic activity of the constitutive nitric oxide synthase (cNOS), whereas protein levels of the three isoforms of NOS remained unchanged [98]. Mice motor disturbances (rigidity) induced by the MPP+ model and associated with dopamine depletion were also blocked by CuSO_4_ [99]. Nowadays, our group is performing a clinical trial in PD patients under a 10 mg copper sulfate supplement b.i.d. controlled with placebo.

## 5. CONCLUSIONS

In conclusion, let us first refer to levodopa therapy. This is the most powerful replacement treatment and, therefore, the gold standard in PD therapeutics, but the following dilemma remains unsolved: “Is it toxic or neuroprotective?” In our experience, we have elements that argue both in favor and against the toxicity of levodopa. For example, the enhanced production of hydrogen peroxide and the subsequent deleterious hydroxyl (^**.**^OH) free radical, *via* the Fenton reaction, in presence of transition metals, is a consequence of the excessive catabolism of this monoamine. On the other hand, under this treatment, the activity of two protective enzymes Cu/Zn SOD and Cp/ferroxidase are significantly increased [95]. Also, levodopa is responsible for the remarkable increase in survival and quality of life in PD subjects. The principal objective of the ELLDOPA study was to analyze rates of progression of PD by taking the mean of the changes in the UPDRS in patients under different doses of levodopa or placebo [100]. The clinical benefit under levodopa at week 42 was marked and dose-dependent [101]. An external analysis also showed that a dose-related increased rate of progression in Parkinson's disease, obscured by symptomatic benefit, is very unlikely [102]. Nevertheless, beta-CIT uptake in the striatum of those patients was proportionally decreased with the dose of levodopa [103]. The doubt is still pending when faced with the possible interpretations of these results, since beta-CIT uptake could be decreased by neuronal loss or by the proper pharmacological effect of levodopa. But the actual tendency in neurological practice focuses on the conservation of the clinical benefits of levodopa by adjusting the dose to fit the needs of the patient, trying to decrease the cumulative dose and the consequent motor complications, and by adding a personalized combination of a dopamine agonist and other potentially neuroprotective medication. The recent ADAGIO study, through the delayed start design testing putative neuroprotective therapy and avoiding symptomatic confound, is solid evidence for an earliest use of rasagiline. In the horizon of PD treatment, appear large amounts of agents already showing excellent results in preclinical and phase I studies [104, 105]. A sample of this is shown in Table **2**. Some of these compounds are destined to fail in clinical phases [106, 107]. New strategies to assay neuroprotective properties are a burning need in human subjects. In addition, actual neuroimage markers, like other *in vivo* markers, require to be developed to obtain solid criteria for the conceptual framework of cell protection in clinical trials.

## Figures and Tables

**Table 1.** Potential Neuroprotective Agents Selected by the CINAPS Group as Good Candidates for Clinical Trials Phase II and III

Compounds Prioritized and Presented in that Order by the CINAPS Group	Background, Further Studied Mechanisms and Present State	References [11]
**Caffeine**	Epidemiological studies Adenosine A_2A_ receptor antagonist	[27] [30, 40]
**Coenzyme Q10 (ubidecarenone)**	Antioxidant and mitochondrial enhancer. Improves both, safety and efficacy at higher doses	[50] [51, 53,54]
**Creatine**	Mitochondrial stabilizer and ATP synthesis enhancer. Good clinical results	[49-50] [48]
**Estrogen**	Epidemiological studies Transcription or signaling	[37] [33-35]
**GM-1 ganglioside**	Neurotrophic enhancer Low bioavailability when peripherally administered.	[57-59]
**Minocycline**	Anti-inflammatory and antiapoptotic actions through caspases 1 and 3 inhibition. Phase II clinical trial.	[41-43, 46] [48]
**Nicotine**	Epidemiological studies reveal decreased risk of PD in tobacco smokers.	
**GPI 1485**	Neuroimmunophilin ligand	[55-56]
**Rasagiline**	MAO-B inhibitor (antioxidant/ antiapoptotic) TEMPO study, ADAGIO study	[21-22] [24-26]
**Selegiline**	MAO-B inhibitor (antioxidant/ antiapoptotic) DATATOP cohort	[14] [18]
**Ropirinole**	Dopamine D2/D3 receptor agonist that slows the rate of decline of functional markers	[68]
**Pramipexole**	Dopamine D2/D3 receptor agonist. Enhances dopamine vesicular trafficking	[4, 66-67]

**Table 2.** Other Agents Currently Used in Clinical Practice, or Reaching Clinical Phases of their Development, with a Neuroprotective Focus

Compounds	Mechanisms	References
**Amantadine** **Memantine**	Both, uncompetitive NMDA* antagonists and, therefore, antiexcitotoxic compounds. Amantadine is indicated in PD tremor and dyskinesias, whereas memantine is used in degenerative dementiae. Studies in PD neuroprotection are pending	[85-86]
**Lamotrigine** **Levetiracetam** **Riluzole**	AED* Decreased glutamate mediated excitotoxicity. Riluzole that increases life span in ALS patients did not have a significant effect on survival or rate of functional deterioration in Parkinson plus syndromes.	[23] [107]
**Talampanel**	Antagonism of excitatory amino acid receptors (AMPA*).	[104]
**Istradefylline**	Adenosine A_2A_ receptor antagonist.	[28, 31]
**Resveratrol** **Green tea polyphenols**	SIRT 1 histone deacetylase enhancer. diverse molecular signaling pathways participating in the neuroprotective activity	[81-82] Approved clinical trial now recruiting PD patients
**Safinamide**	MAO-B* inhibitor that also acts on glutamate pathway	[79-80]
**Ebselen** **Thiol containing antioxidants**	Mimics glutathione peroxidase activity. Avoids glutathione depletion within CNS.	Clinical trials in stroke
**CEP-1347**	Antiapoptotic through mixed-lineage kinases inhibition Failed in PD	[104-105]
**GDNF**	Neurotrophic factor. Methods for the delivery of GDNF* into basal ganglia are discussed in the referenced review	[104]
**GAD gene delivery**	The first illustration of gene therapy reaching clinical phase I by the mean of a viral vector implanted in the STN*	[104-105]
**Zonisamide**	Inhibition of DA-induced quinone toxicity. Increased levels of glutathione and manganese superoxide dismutase expression. Moderate inhibition of MAO-B activity. Inhibition of voltage-dependent sodium and T-type calcium channels.	[78]

* NMDA= N-methyl D-aspartate; AMPA= α-amino-3-hydroxy-5-methyl-4-isoxazolepropionic acid; AED= antiepileptic drugs; MAO-B= Type B Monoamine Oxidase; GAD= Glutamate Decarboxylase; STN= Subthalamic Nucleus; GDNF= Glial Cell Line-Derived Neurotrophic Factor.
